# Dimethylimidazolium-Functionalized Polybenzimidazole and Its Organic–Inorganic Hybrid Membranes for Anion Exchange Membrane Fuel Cells

**DOI:** 10.3390/polym13172864

**Published:** 2021-08-26

**Authors:** Li-Cheng Jheng, Cheng-Wei Cheng, Ko-Shan Ho, Steve Lien-Chung Hsu, Chung-Yen Hsu, Bi-Yun Lin, Tsung-Han Ho

**Affiliations:** 1 Department of Chemical and Materials Engineering, National Kaohsiung University of Science and Technology, Kaohsiung 80778, Taiwan; lcjheng@nkust.edu.tw (L.-C.J.); hks@nkust.edu.tw (K.-S.H.); j841216@gmail.com (C.-Y.H.); 2 Department of Materials Science and Engineering, National Cheng Kung University, Tainan 70101, Taiwan; kevin850427@gmail.com; 3 Instrument Center of National Cheng Kung University, Tainan 70101, Taiwan; z7710011@email.ncku.edu.tw

**Keywords:** polybenzimidazole, anion exchange membrane, fuel cells, dimethylimidazolium, organic–inorganic hybrid membrane

## Abstract

A quaternized polybenzimidazole (PBI) membrane was synthesized by grafting a dimethylimidazolium end-capped side chain onto PBI. The organic–inorganic hybrid membrane of the quaternized PBI was prepared via a silane-induced crosslinking process with triethoxysilylpropyl dimethylimidazolium chloride. The chemical structure and membrane morphology were characterized using NMR, FTIR, TGA, SEM, EDX, AFM, SAXS, and XPS techniques. Compared with the pristine membrane of dimethylimidazolium-functionalized PBI, its hybrid membrane exhibited a lower swelling ratio, higher mechanical strength, and better oxidative stability. However, the morphology of hydrophilic/hydrophobic phase separation, which facilitates the ion transport along hydrophilic channels, only successfully developed in the pristine membrane. As a result, the hydroxide conductivity of the pristine membrane (5.02 × 10^−2^ S cm^−1^ at 80 °C) was measured higher than that of the hybrid membrane (2.22 × 10^−2^ S cm^−1^ at 80 °C). The hydroxide conductivity and tensile results suggested that both membranes had good alkaline stability in 2M KOH solution at 80 °C. Furthermore, the maximum power densities of the pristine and hybrid membranes of dimethylimidazolium-functionalized PBI reached 241 mW cm^−2^ and 152 mW cm^−2^ at 60 °C, respectively. The fuel cell performance result demonstrates that these two membranes are promising as AEMs for fuel cell applications.

## 1. Introduction

Polymer electrolyte membrane fuel cells are one type of fuel cell suitable for portable and transportation applications, which feature quick start-up, low operation temperature, low cost, high efficiency, and so on [1,2]. Depending on the polymer electrolyte membrane, this type of fuel cell can be further divided into proton exchange membrane fuel cell (PEMFC) [3,4] and anion exchange membrane fuel cell (AEMFC) [5,6]. AEMFC has been attracted considerable attention in the recent decade due to several advantages over PEMFC, including (1) faster oxygen reduction reaction (ORR) under the basic condition that allows the catalyst at the cathode to employ platinum-free metal or low-platinum catalysts [7,8,9], (2) negligible fuel crossover owing to the hydroxide transport direction opposite to the direction of liquid fuel’s crossover (e.g., methanol) [10,11], as well as (3) minimized corrosion problems in alkaline environments [2,12].

Although AEMFC is one of the most promising platinum-free fuel cells for power conversion applications, its core component, anion exchange membrane (AEM), still faces several challenges, such as insufficient hydroxide conductivity, poor dimensional stability, and inadequate alkaline stability [13]. Generally, there is a trade-off relationship between the ionic conductivity and dimensional stability of an AEM [14]. For most AEMs, high IEC leads to an increased hydroxide conductivity but impaired dimensional stability due to excessive water-uptake [15]. Additionally, most commonly used cationic groups (e.g., quaternary ammonium, imidazolium, benzimidazolium, sulfonium) for AEMs are unstable and susceptible to degrade via nucleophilic displacement or Hofmann elimination under high pH conditions especially at temperatures above 60 °C [16,17]. Furthermore, main chain type AEMs constituted by polymers bearing cationic groups in the main chain may accelerate the alkaline degradation of cationic groups because of the electron-withdrawing effect of the benzene ring particularly for polymers containing aryl ether moieties [18]. To solve these addressed problems simultaneously, fabricating hydrophilic ion transport channels in the AEMs through the ionic clustering formation and the hydrophilic/hydrophobic micro-phase separations has been suggested as a feasible strategy [13].

Preparing side chain type AEMs (or called comb-shaped polymer based AEMs), which is composed of flexible long side chains with hydrophilic cationic groups covalently grafted onto the polymer backbone, is one of efficient ways to develop hydrophilic/hydrophobic micro-phase separations due to the dissimilarity between the side chain and polymer backbone [7]. Recently, a variety of polymer backbones have been investigated for developing side chain type AEMs with high performance, such as polyphenylene oxide (PPO) [19,20], polyarylene ether sulfone (PAES) [21,22], polyether ether ketone (PEEK) [23], polybenzimidazole (PBI) [24,25,26], polynorbornene [27,28], polystyrene-based copolymer [29,30]. However, aryl ether-containing based AEMs might suffer from alkaline degradations of polymer backbone in alkaline media [31]. AEMs based on aliphatic polymer backbones may lack thermal stability and mechanical strength. In contrast, PBI is a basic polymer with excellent thermal stability, high mechanical strength, and low water uptake. PBI even can be doped with a high concentration of KOH solution to obtain heterogeneous AEMs, namely KOH-doped PBI [32,33]. Therefore, PBI is considered a good candidate as the polymer backbone alkaline stable for AEMs [34].

Aside from the development of hydrophilic ion transport channels, crosslinking is another common method capable of improving dimensional stability, mechanical strength and chemical stability of AEMs. However, the AEM’s ion exchange capacity (IEC) may be decreased due to the incorporation of the crosslinker into it. Therefore, some research efforts have been made to develop silane-based quaternized crosslinkers consisting of cationic groups and silane groups that can undergo a sol–gel process to form organic–inorganic hybrid networks and provide additional anion conducting sites within the crosslinked AEMs [35,36,37,38,39]. For example, Li and his coworkers prepared a polysulfone based AEM crosslinked by 4,4′-oxydiphenylguanidinium-bridged-silsesquioxane (ODGBS) with an IEC of 2.47 mmol g^−1^ that exhibited a high hydroxide conductivity up to 2.5 × 10^−2^ S cm^−1^ at 30 °C [37]. In the work of Wang et al., their sol–gel-derived silica/PVA-Py hybrid AEMs had IEC ranging from 0.54 mmol g^−1^ to 0.92 mmol g^−1^ depending on the added amount of the quaternary ammonium-functionalized crosslinker [39].

Our previous study demonstrated that the side chain type quaternized PBI with methylimidazolium end-capped groups exhibited better alkaline stability and ionic conductivity compared with the main chain type quaternized PBI [40]. Recently, Lin and Ding et al. reported that the hydrophilic/hydrophobic micro-phase separation morphology can successfully form in the AEMs based on side chain type quaternized PBI containing dimethylimidazolium cations [24]. Wu and He et al. studied the influence of grafting strategies on the hydroxide conductivity and fuel cell performance for the AEMs based on side chain type quaternized PBI with quaternary ammounium end-capped groups [25]. Among these cations, dimethylimidazolium is expected to be more alkaline stable. The reason is that the C2-substituted methyl group of dimethylimidazolium can increase the steric interference and replace the acidic C2 protons to weaken the hydroxide attack, suggested by Xu and Yan’s previous studies [41,42]. 

In the present work, we attempt to synthesize a new type of quaternized PBI-based AEMs using a combined strategy of quaternized side chain grafting and silane-induced crosslinking. A silane-based quaternized crosslinker (triethoxysilylpropyl dimethylimidazolium chloride, [SPDMIm] [Cl]) and a long flexible side chain containing dimethylimidazolium end group (bromohexyl dimethylimidazolium bromide, [BrHDMIm] [Br]) were synthesized and characterized. A side chain type dimethylimidazolium-functionalized PBI pristine membrane and its organic–inorganic hybrid membrane were prepared as AEMs for fuel cells. The morphology difference between the pristine and hybrid membranes was investigated. Membrane properties of AEMs including ion exchange capacity, water uptake, swelling ratio, hydroxide conductivity, and mechanical strength were measured. The thermal, alkaline, and oxidative stabilities of the prepared AEMs were evaluated. Furthermore, the fuel cell performance tests were carried out to confirm that the pristine and organic–inorganic hybrid membranes of dimethylimidazolium-functionalized PBI promising as AEMs for AEMFC applications.

## 2. Materials and Methods

### 2.1. Materials

3,3′-Diaminobenzidine (DAB), sodium hydride (NaH) (dry, 90%), polyphosphoric acid (PPA), and N,N-dimethylacetamide (DMAc) were received from Sigma-Aldrich. Phosphorous pentoxide (P_2_O_5_) and sodium hydroxide (NaOH) were provided by SHOWA and Alfa Aesar, respectively. 2,2-bis(4-carboxyphenyl)hexafluoropropane (6FDA) and 3-chloropropyltriethoxysilane were purchased from TCI. 3-chloropropyltrimethoxysilane (CPTMOS), 1,2-dimethylimidazole, 1,6-dibromohexane, N,N-dimethylacetamide (DMF), and dimethylsulfoxide (DMSO) (extra dry) were supplied from Acros Organics. All the reagents were ACS grade and used as received without further purifications. Nitrogen, hydrogen, and oxygen gases with a high purity of greater than 99.99% were provided from a local manufacturer (Jing-Shang gas).

### 2.2. Synthesis of Polybenzimidazole (PBI)

Polybenzimidazole (PBI) employed in this work was hexafluoroisopropylidene containing PBI, namely 6FPBI, synthesized from DAB and 6FDA in phosphoric acid containing extra P_2_O_5_. The detailed procedures followed our previous work [43,44]. The inherent viscosity of the synthesized PBI was measured to be 2.1 dL/g using a Ubbelohde tube viscometer in DMAc at 30 °C.

### 2.3. Synthesis of Bromohexyl Dimethylimidazolium Bromide ([BrHDMIm] [Br])

1,2-Dimethylimidazole (50 mmol), 1,6-dibromohexane (60 mmol), and acetonitrile (50 mL) were added to a three-necked flask equipped with a condenser. The mixture was stirred with a magnetic stirrer at 60 °C for 15 h under a nitrogen atmosphere. A viscous liquid formed after the mixture was added dropwise into an excess amount of diethyl ether under stirring. Then, the resulting viscous liquid was washed with ethyl acetate three times and was subsequently dried by evaporation under vacuum at 90 °C for 24 h. After that, a brown viscous liquid (BrHDMIm; Br) was obtained.

### 2.4. Synthesis of Triethoxysilylpropyl Dimethylimidazolium Chloride ([SPDMIm] [Cl])

1,2-Dimethylimidazole (50 mmol) and 3-chloropropyltrimethoxysilane (CPTMOS) (50 mmol) were dissolved in DMF (15 mL) in a three-necked flask equipped with a condenser. The mixture was stirred under nitrogen at 80 °C for 24 h. After that, the mixture was poured into diethyl ether to obtain a viscous liquid. The resulting liquid was collected and washed with diethyl ether twice. After the resulting liquid was dried under vacuum at 75 °C for 24 h, a light yellow viscous liquid [SPDMIm] [Cl] was obtained. 

### 2.5. Synthesis of Dimethylimidazolium-Functionalized Polybenzimidazole (PBI-DIm)

PBI were dried in a vacuum oven at 100˚C overnight in advance. PBI (2 mmol) was dissolved in anhydrous DMSO (50 mL) under nitrogen at 80 °C in a three-necked flask equipped with a condenser. After cooling to room temperature, an excess NaH (4.8 mmol) was added to the PBI solution. The solution kept stirring at 80 °C for about 4 h until the bubbling stopped. After that, an excess amount of [BrHDMIm] [Br] (6 mmol) was added to the solution. The mixture was stirred under nitrogen at 80 °C for 24 h. The resulting polymer was precipitated by pouring the mixture into an excess amount of acetone. A dimethylimidazolium-functionalized PBI (PBI-DIm) was obtained after the resulting polymer was washed with deionized water and dried in a vacuum oven at 90 °C for 24 h. Meanwhile, the functionalization degree of the synthesized PBI-DIm was determined to be 1.93 pendant dimethylimidazolium groups per repeated unit by 1H NMR analysis.

### 2.6. Fabrication of PBI-DIm Pristine Membranes and PBI-DIm-Si Hybrid Membranes

A certain amount of PBI-DIm powders was dissolved in DMSO (40 mL) to make a 2 wt% polymer solution. To form PBI-DIm pristine membranes, the PBI-DIm solution (20 mL) was directly cast on a glass substrate and dried under a reduced pressure at 80 °C for 12 h followed by a vacuum at 110 °C for 12 h. After peeling off the pristine membranes from the substrate, they were washed with methanol to remove any residual DMSO solvent and dried at 60 °C for 2 h.

For preparing PBI-DIm-Si hybrid membranes, predetermined amounts of CPTMOS and [SPDMIm] [Cl] were added into the 2 wt% PBI-DIm solution (20 mL) in a sealed vial. The molar ratio of CPTMOS/[SPDMIm] [Cl]/PBI-DIm was fixed to be 0.1/0.1/1. A few drops of diethylamine and deionized water were added to the solution. Then, it was stirred at 50 °C for 24 h to promote the hydrolysis of silane groups. The solution was subsequently poured into a Petri dish with a cover and placed in an oven at 110 °C for 6 h. After the cover of the Petri dish was taken away and the solution was dried under a reduced pressure at 110 °C for 6 h followed by a vacuum at 110 °C for 12 h, the PBI-DIm-Si hybrid membranes were formed. After peeling off the hybrid membranes from the Petri dish, they were washed with methanol to remove any residual DMSO solvent and dried at 60 °C for 2 h.

### 2.7. Characterization

^1^H nuclear magnetic resonance (NMR) spectra for compound and polymer samples dissolved in DMSO-d_6_ were obtained on a Bruker AVANCE 600 MHz spectrometer with a frequency of 600.3 MHz. Fourier transform infrared (FT-IR) spectra were recorded on a JASCO FT/IR 4600 spectrometer by scanning 16 times in a transmittance mode and a wavelength range between 400 cm^−1^ and 2000 cm^−1^ with a resolution of 2 cm^−1^. Thermogravimetric analysis (TGA) was recorded on a TA instrument model 2050 at a heating rate of 10 °C/min from 100 °C to 800 °C with a 20 mL/min nitrogen flow. Thermomechanical analysis was performed to measure the in-plane coefficients of thermal expansion (CTEs) and glass transition temperatures (Tgs) of the membrane samples on a TA Instrument Q400EM. Atomic force microscopy (AFM) phase images were taken by mapping in a taping mode on a Bruckner Dimension ICON SPM at ambient temperature for observing the surface of the prepared membranes in their dry state. X-ray photoelectron spectroscopy (XPS) was conducted on a ULVAC PHI5000 VersaProbe instrument with a monochromatized Al Kα X-ray source generated by an electron beam (24.7 W). The XPSPEAK software version 4.1 was employed to deconvolute spectra into component peaks using a curve fitting method based on Gaussian Lorentzian sum functions. The background of the spectra was subtracted using the Shirley method. A scanning electron microscope (SEM) JEOL JSM-5610 combined with an energy dispersive X-ray spectroscopy (EDX) system INCA supplied by Oxford Instruments was employed to carry out elemental mapping characterization. The cross-sectional SEM images of the membrane samples were taken by a high-resolution scanning electron microscope (HE-SEM), ITACHI SU8000. Small angle X-ray scattering (SAXS) analysis was performed on a Bruker NANOSTAR U SYSTEM equipped with a VANTEC-2000 detector.

### 2.8. Measurements

Ion exchange capacity (IEC) of a membrane was measured by the Mohr titration method. The membrane was immersed in 1 M NaCl solution at RT for 24 h to replace all anions in the membrane with Cl^−^ ions for 24 h. After washing with deionized water to remove any residual ions on the surface, the membrane was dried in a vacuum oven at 100 °C for 3 h and its dry weight was recorded. Afterward, the dry membrane was immersed in 1 M Na_2_SO_4_ solution at RT for 24 h for releasing Cl^−^ ions to the solution. The amount of released Cl^−^ ions was titrated with 0.1 M AgNO_3_ using K_2_CrO_4_ as a colorimetric indicator. The experimental IEC is calculated according to the following equation:

(1) $$\mathrm{IEC}=\frac{V_{AgNO_{3}}\times C_{AgNO_{3}}}{m_{\mathrm{dry}}}$$

where V*_AgNO3_* and C*_AgNO3_* are the consumed volume and the concentration of AgNO_3_ solution. m_dry_ is the weight of the dry membrane in Cl^−^ form.

The water uptake (WU) and swelling ratio (SR) of a membrane were measured according to its weight and length changes in between the wet and dry states, respectively. Before measurement, the membrane was immersed in 1 M KOH solution at RT for 24 h to complete ion exchange, followed by washing with deionized water. After the membrane was picked out from the vial and wiped with tissue paper to remove surface water, the weight and length of the wet membrane (*W_wet_* and *L_wet_*) were measured. Then, it was dried in a vacuum oven at 100 °C for 3 h and followed by measuring its weight and length in the dry state (*W_dr_*_y_ and *L_dry_*). The membrane’s WU and SR is calculated as follows:

(2) $$\mathrm{WU}=\frac{W_{wet}-W_{dry}}{W_{dry}}$$

(3) $$\mathrm{SR}=\frac{L_{wet}-L_{dry}}{L_{dry}}$$

The hydration number of a membrane (λ) referring to the number of absorbed water molecules around each dimethylimidazolium group was calculated as follows.

(4) $$\lambda =\frac{10\times WU\left(\%\right)}{18\times IEC}$$

The insoluble solid content of a membrane was determined by the dry weight change of a membrane before and after re-dissolved in DMSO. A membrane sample was re-dissolved in DMSO at RT for 72 h under gentle stirring. Then, the membrane was immersed in excess methanol for 12 h to leach DMSO from it. The resulting membrane was dried under vacuum at 60 °C for 12 h. The insoluble solid content of the membrane can be determined according to the equation below.

(5) $$\mathrm{Insoluble}\;\mathrm{solid}\;\mathrm{content}\;\frac{W_{dry}-W_{\mathrm{dry}.re-dissolved}}{W_{dry}}$$

The terms *W_dry_* and *W_dry,re-dissolved_* are referred to as the weight of the dry membrane before and after being re-dissolved in DMSO, respectively.

### 2.9. Hydroxide Conductivity and Alkaline Stability

The hydroxide conductivity of a membrane was measured using the electrochemical impedance spectroscopy method, which was conducted on an Autolab PGSTAT128N impedance analyzer connected to a 4-electrode cell (BekkTech BT-112) over the frequency range from 10^2^ Hz to 10^5^ Hz with an alternating voltage amplitude of 10 mV. Before measurement, the membrane was immersed in 1 M KOH solution at RT for 24 h to complete ion exchange, followed by washing with deionized water. The membrane sample in a 30 mm × 4.5 mm dimension was inserted in the 4-electrode cell, which was placed in N_2_-saturated deionized water in a sealed flask. The in-plane hydroxide conductivity was calculated by the following equation.

(6) $$\sigma =\frac{L}{A\times R_{\Omega }}$$

where *σ* is the in-plane hydroxide conductivity of the fully hydrated membrane (S cm^−1^). *L* is the length between the two voltage measuring probes (0.425 cm). *A* is the cross-sectional area perpendicular to the current flow (cm^2^). *R*_Ω_ is the ohmic resistance of the membrane, obtained from the approximate value of the real impedance-axis intercept in a Nyquist plot.

The alkaline stability of the membrane was evaluated by the change in hydroxide conductivity during an alkaline treatment that is carried out by immersing the OH^−^ form membrane in 2 M KOH at 80 °C for 120 h. Before each hydroxide conductivity measurement, the membrane sample was washed with deionized water several times and kept in N_2_-saturated deionized water for at least 12 h.

### 2.10. Mechanical Properties and Oxidative Stability

Mechanical properties of a membrane sample were examined on a Shimadzu AG-SI universal testing machine at a 20 mm/min tensile testing speed under ambient conditions. The samples were prepared at 50 mm in length, 4.5 mm in width, and approximately 30–40 µm in thickness. Each result is the average value for at least six samples. The oxidative stability of a membrane was evaluated using the Fenton test. The membrane was immersed in a 3 wt% hydrogen peroxide aqueous solution containing 4 ppm Fe^2+^ at 80 °C. The remaining weight of the degraded membrane in the dry state was recorded every 2 h.

### 2.11. Fuel Cell Tests

A catalyst ink was prepared by mixing a Pt/C catalyst (VULCAN^®^ XC-72R/Cabot, College Station, Texas, TX, USA, 40 wt% Pt) with an ionomer solution (Sustainion^®^ XB-7/Dioxide Materials, Boca Raton, FL, USA, alkaline ionomer 5% in ethanol) and an aqueous isopropyl alcohol solution. The solid content ratio of the ionomer to the Pt/C catalyst was 1:4. The catalyst ink was sonicated for 5 min in an ice bath with a probe-type sonicator. Subsequently, it was coated onto carbon papers (N1S1007/QuinTech Indiana, PA, USA) on a hot plate by drop-casting to prepare gas diffusion electrodes (GDEs) at a Pt loading of 0.8 mg cm^−1^. Three AEMs in OH form including the PBI-DIm membrane, PBI-DIm-Si hybrid membrane, and the commercially available AEM Sustainion^®^ X37-RT (purchased from Dioxide Materials, Inc.) were individually sandwiched between two GDEs without hot-pressing to fabricate their membrane electrode assemblies (MEAs). The effective area of the MEAs was 4 cm^2^. The fuel cell performance of the MEA at 60 °C under ambient pressure was examined in a fuel cell test system (Tension Energy, Inc., Zhubei, TaiWan) equipped with an electronic load unit controller. The flow rates of the humidified hydrogen and oxygen were controlled to be 50 mL min^−1^ and 100 mL min^−1^, respectively.

## 3. Results and Discussion

### 3.1. Synthesis and Characterization

**Scheme 1:**
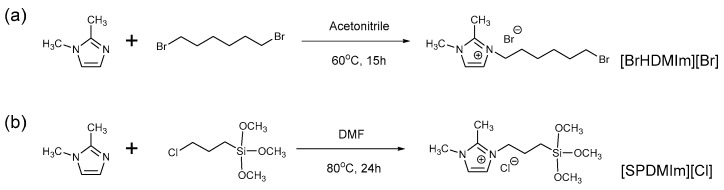
Synthetic routes of (**a**) [BrHDMIm] [Br] and (**b**) [SPDMIm] [Cl].

**Scheme 2:**
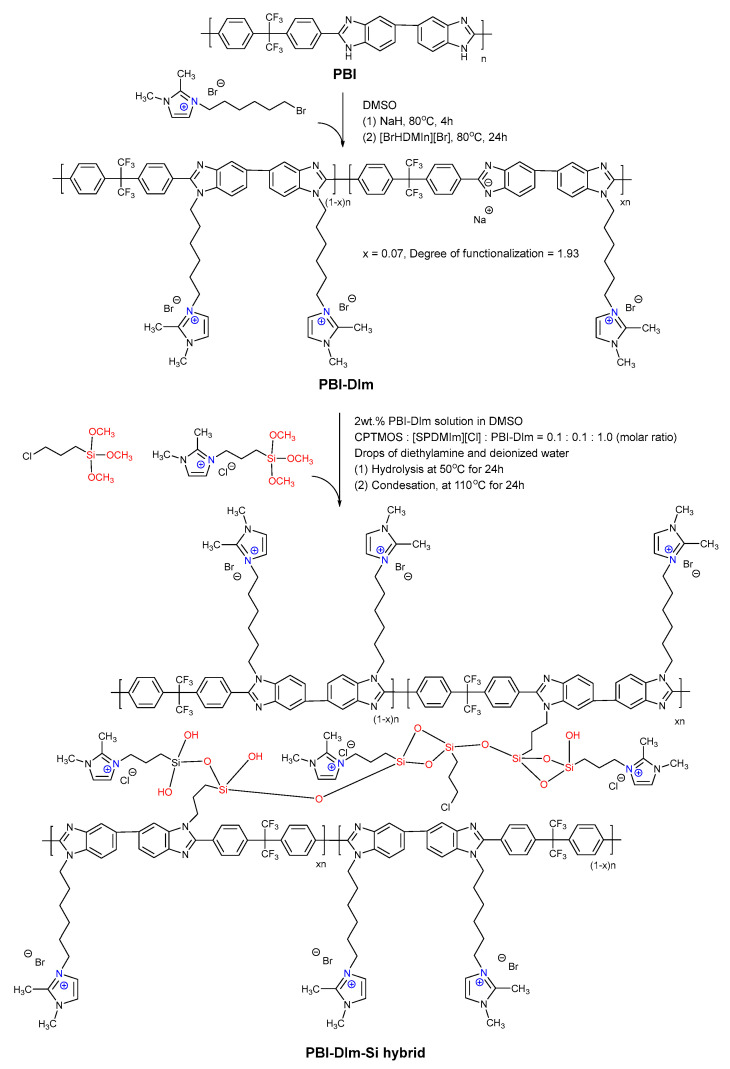
Synthetic route of PBI-DIm and PBI-DIm-Si hybrid.

In this work, we synthesized two dimethylimidazolium based ionic liquids [BrHDMIm] [Br] and [SPDMIm] [Cl] by the Menshutkin reaction as shown in Scheme 1. [BrHDMIm] [Br] was grafted onto PBI by the S_N_2 reaction to obtain side chain type quaternized PBI, denoted PBI-DIm. The functionalization degree of PBI-DIm was determined to be 1.93 (dimethylimidazolium groups per repeated unit) by ^1^H NMR integration analysis. The functionalization degree of less than 2 implied that no alkaline unstable benzimidazolium groups in the polymer main chain formed. Meanwhile, PBI-DIm still had some sites for the further grafting of silane pendant groups (CPTMOS). As presented in Scheme 2, the appropriate amount of [SPDMIm] [Cl] and CPTMOS was introduced into PBI-DIm to carry out a crosslinking reaction involving hydrolysis and condensation processes for preparing a quaternized PBI organic–inorganic hybrid (PBI-DIm-Si hybrid). This silane-induced crosslinking reaction underwent along with the membrane casting process in order to obtain homogeneous membranes.

**Figure 1:**
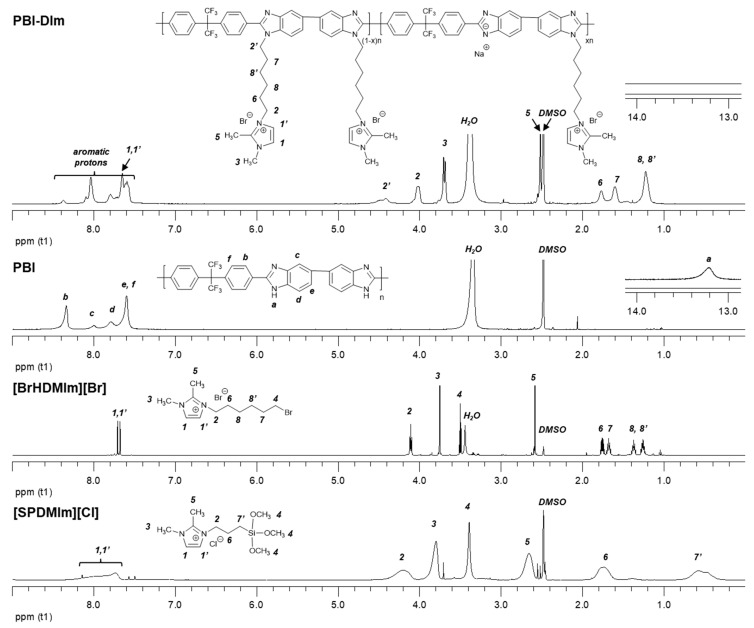
^1^H NMR spectra of [SPDMIm] [Cl], [BrHDMIm] [Br], PBI, and PBI-DIm.

The chemical structures of [BrHDMIm] [Br], [SPDMIm] [Cl], PBI, and PBI-DIm were confirmed by ^1^H NMR analysis as shown in Figure 1. All the signals of PBI were in accordance with those reported in the previous studies [40]. The characteristic signals (H_2_) corresponding to the aliphatic methylene protons adjacent to dimthylimidazolium for [BrHDMIm] [mBr], [SPDMIm] [Cl], and PBI-DIm appeared at the chemical shift at 4.10 ppm, 4.20 ppm, and 4.03 ppm, respectively. Meanwhile, the signals attributed to the methyl protons of dimthylimidazolium moiety (H_3_ and H_5_) for [BrHDMIm] [Br], [SPDMIm] [Cl], and PBI-DIm were located at around 3.7–3.8 ppm and 2.5–2.7 ppm. In the PBI-DIm spectrum, we found that an additional signal (H_2’_) at the chemical shift from 4.35 ppm to 4.6 ppm ascribed to the methylene protons nearby the nitrogen of benzimidazole was present, but the signal (H_4_) for [BrHDMIm] [Br] corresponding to the methylene protons adjacent to bromine at 3.51 ppm was absent. This finding suggested that the grafting of [BrHDMIm] [Br] onto PBI backbone by covalent bonding has taken place. The other signals (H_6_, H_7_, H_7’_, H_8_, and H_8’_) related to the methylene protons of [BrHDMIm] [Br], [SPDMIm] [Cl], and PBI-DIm were individually assigned in the chemical shift range of 0.3 ppm and 1.9 ppm, as seen in Figure 1.

**Figure 2:**
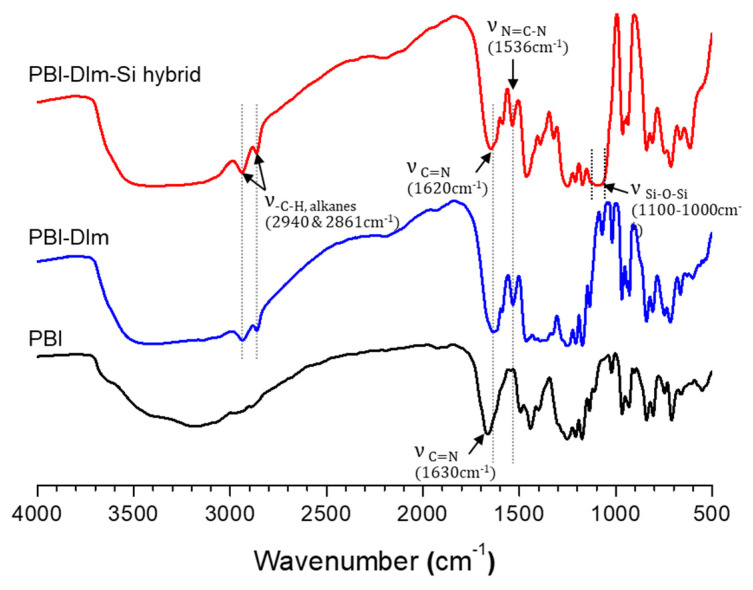
FTIR spectra of PBI, PBI-DIm, and PBI-DIm-Si hybrid membranes.

The FTIR spectra of the PBI, PBI-DIm, and PBI-DIm-Si hybrid membranes were compared in Figure 2. The characteristic absorption band corresponding to the N=C–N stretching vibration of the imidazolium moiety was observed at 1536 cm^−1^ in both spectra of PBI-DIm and PBI-DIm-Si hybrid [45]. Meanwhile, the absorption band attributed to the C=N stretching vibration of the benzimidazole moiety was noticed to shift from 1630 cm^−1^ (for PBI) to 1620 cm^−1^ (for PBI-DIm and PBI-DIm-Si hybrid). This slight shift may arise from the grafting of the side chains onto PBI, in line with the previous finding [40]. The alkane C–H stretching vibrations of methylene and methyl groups belonging to the dimethylimidazolium side chains of PBI-DIm and PBI-DIm-Si hybrid resulted in two absorption bands at 2861 cm^−1^ and 2940 cm^−1^. Moreover, the band due to the asymmetric Si–O–Si stretching vibration usually appears in the region between 1130 cm^−1^ and 1030 cm^−1^ can be found only in the spectrum of PBI-DIm-Si hybrid [46]. This finding indicated that the hydrolysis and condensation occurred during the sol–gel process.

**Figure 3:**
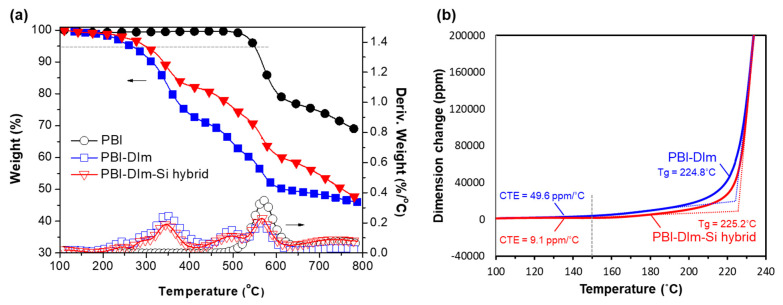
(**a**) Thermograms of PBI, PBI-Dim, and PBI-DIm-Si hybrid membranes in a temperature range from 100 °C to 800 °C under a nitrogen atmosphere. (**b**) Dimension changes of the PBI-DIm and PBI-DIm-Si hybrid membranes as a function of temperature.

Compared with PBI polymer backbone, imidazolium cations are relatively unstable and possible to undergo thermal degradation when the temperature exceeds 250 °C [47,48]. Figure 3a presented the TGA curves of the PBI, PBI-DIm, and PBI-DIm-Si hybrid membranes recorded from 100 °C to 800 °C. As expected, the weight loss in the temperature range approximately between 250 °C and 400 °C due to the decomposition of dimethylimidazolium side chains was detected for PBI-DIm and PBI-DIm-Si hybrid. The weight loss at the temperature above 500 °C attributed to the decomposition of the PBI main chains can be found for the three thermograms. Moreover, the decomposition temperature of 5 wt.% weigh loss (T_d5_) of PBI-DIm-Si hybrid was noticed to be slightly higher than that of the PBI-DIm.

A thermomechanical analysis (TMA) can be used to determine the glass transition temperatures (Tg) and the coefficient of thermal expansion (CTE) of a membrane. Figure 3b shows the dimension changes of the PBI-DIm and PBI-DIm-Si hybrid membranes as a function of temperature. Notably, the Tgs of these two membranes were almost the same (224.8 °C and 225.2 °C), but the difference between their CTEs was considerable. The CTE of the PBI-DIm-Si hybrid membrane in the temperature range from 100 °C to 150 °C was determined to be 9.1 ppm/°C, much lower than that of PBI-DIm membrane (49.6 ppm/°C) and the CTEs of most of the semi-crystalline polymers [49]. This finding suggested that the organic–inorganic hybrid structure is beneficial for the dimensional stability of the membrane when the temperature varies below the quaternized PBI’s Tg.

### 3.2. Membrane Morphology

**Figure 4:**
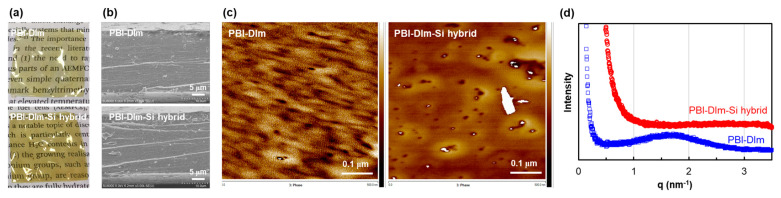
(**a**) Photographs, (**b**) SEM cross-sectional micrographs, (**c**) AFM phase images of the PBI-DIm and PBI-DIm-Si hybrid membranes, and (**d**) SAXS profiles of PBI-DIm and PBI-DIm-Si hybrid membranes.

It can be seen that both the appearance of PBI-DIm and PBI-DIm-Si hybrid membranes were transparent and homogeneous (Figure 4a). Additionally, quite smooth and dense surfaces of these two membranes were observed from their SEM cross-sectional micrographs in Figure 4b, revealing that their gas separation for hydrogen and oxygen gases would function well.

AFM in a tapping mode was employed further to characterize the surface morphology of the as-prepared membranes. For side chain type AEMs, the immiscibility between hydrophilic side chains and hydrophobic polymer backbone may contribute to microphase separations and fabrication of hydrophilic channels beneficial for ion transports^19^. As shown in Figure 4c, we observed a distinguishable hydrophilic/hydrophobic phase separation in the AFM phase image of the PBI-DIm membrane in a scan area of 500 nm × 500 nm. The darker regions represented hydrophilic domains constructed by the ionic clusters of the dimethylimidazolium side chains, whereas the brighter regions were the hydrophobic domains comprising the PBI main chain^41^. In contrast, the phase separation was unclear, and many white spots were present in the PBI-DIm-Si hybrid membrane. The hydrophilic/hydrophobic phase separations appeared to be interrupted by the organic–inorganic hybrid networks. The presence of white spots would result from the formation of the silica-based component during the sol-gel process.

The SAXS diffraction technique can be used to analyze the average ionic cluster dimension in anion exchange membranes [50]. As shown in Figure 4d, a broad ionic peak can be observed from the SAXS diffraction profile of PBI-DIm, indicating that an ordered structure due to the hydrophilic/hydrophobic phase separation has been developed within the quaternized PBI pristine membrane. The maximum scattering peak position was approximately at 1.66 nm^−1^, which corresponded to the interdomain Bragg spacing of 3.79 nm. Meanwhile, there was no SAXS scattering peak for the PBI-DIm-Si hybrid membrane, in line with the AFM result. Both AFM and SAXS results suggest the aggregation of ionic clusters successfully occurred in the PBI-DIm pristine membrane, which could facilitate the development of the hydrophilic ion transport channels. As a result, it can be expected that the ionic conduction will be faster in the pristine membrane than in the hybrid membrane.

**Figure 5:**
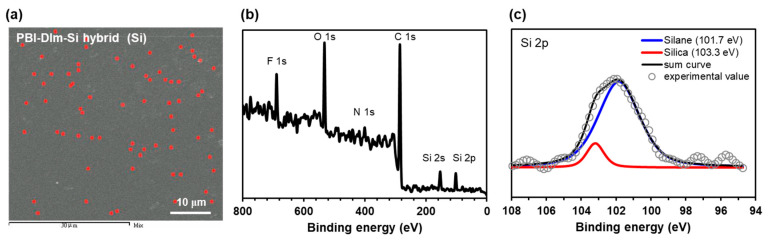
(**a**) EDX elemental mapping (red: silicon), (**b**) XPS full spectrum, and (**c**) high-resolution spectrum of Si 2p core-level peak for the PBI-DIm-Si hybrid membrane.

Using EDX elemental mapping technique (Figure 5a), the silicon element was observed uniformly dispersed within the PBI-DIm-Si hybrid. Moreover, Figure 5b,c show the XPS full spectrum and high-resolution spectrum of Si 2p core-level peak for the PBI-DIm-Si hybrid membrane. The Si 2p peak can be deconvoluted into two sub-peaks at 101.7 eV for silane and 103.3 eV for silica [51], revealing that the partial silicon element was converted from organic silane to inorganic silica. This result confirmed the formation of the silica-based component in the hybrid membrane.

### 3.3. Ion Exchange Capacity, Water Uptake and Swelling Ratio

The experimental IECs of PBI-DIm and PBI-DIm-Si hybrid, measured by the Mohr titration method for their Cl^−^ form membranes, agreed with their theoretical IECs listed in Table 1. Owing to some additional dimthylimidazolium cations coming from (SPDMIm; Cl), the IEC of PBI-DIm-Si hybrid (1.87 mmol g^−1^) was found slightly higher than that of PBI-DIm (1.85 mmol g^−1^) as expected.

The water uptake of an AEM is essential for ion dissociation and ion conduction, which is affected by the IEC and the chemical structure of the polymer. However, excessive water uptake by the membrane would lead to poor dimensional stability and weak mechanical properties [52]. However, the side chain type AEM based on PBI-DIm with high IEC exhibited moderate water uptake (WU = 62.7%) and swelling ratio (SR = 26.7%), owing to the PBI backbone containing hydrophobic hexafluoroisopropylidene moiety. Notably, the WU and SR further declined to 30.9% and 10.0% for the PBI-DIm-Si hybrid membrane. The reason may lie in the fact that organic–inorganic hybrid networks reduced the water affinity of the membrane [53]. Additionally, the swelling ratios of these two membranes were lower than the reported value of Nafion 117 (28%) [54], suggesting that their dimensional stabilities were adequate for fuel cell applications.

The hydration number (λ), determined by the ratio of experimental IEC and WU values, represents the number of water molecules around each cationic group. PBI-DIm had a relatively high hydration number (18.8), about twice higher than that of PBI-DIm-Si hybrid. This result can explain why the hydrophilic channels beneficial for ion conduction were successfully developed in the PBI-DIm membrane (Figure 4c).

**Scheme 3:**
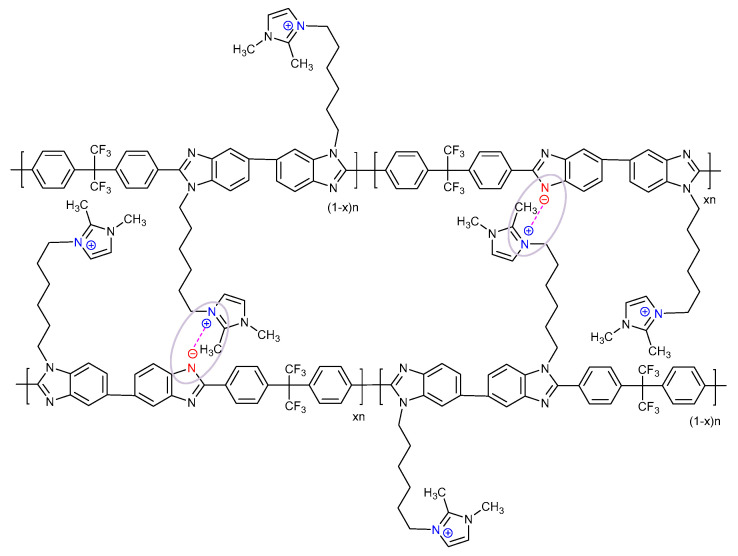
Possible intermolecular interactions between PBI-DIm.

It is noticed that the as-prepared AEMs cannot re-dissolve in the casting solvent DMSO. The intermolecular interactions between dimethylimidazolium cationic groups in the side chain and deprotonated bezimidazole anionic groups in the main chain through ionic attraction, as illustrated in Scheme 3, could be the possible reason for the reduced solubility of the quaternized PBI in DMSO [25,55]. The insoluble solid content of the PBI-DIm and PBI-DIm-Si hybrid membranes were measured to be 62.4 wt% and 78.8 wt%, respectively. As expected, the crosslinking networks of the PBI-DIm-Si hybrid membrane further increase its insoluble gel fraction.

### 3.4. Hydroxide Conductivity and Alkaline Stability

**Figure 6:**
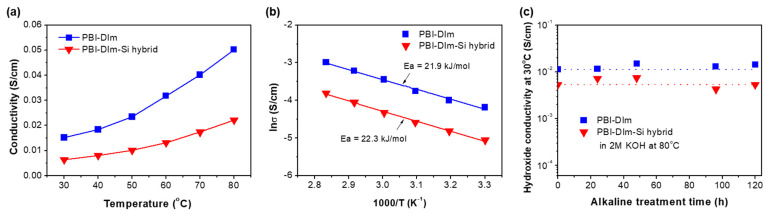
(**a**)Temperature dependence of hydroxide conductivity, (**b**) the Arrhenius plot, and (**c**) hydroxide conductivity measured at 30 °C as a function of time during the alkaline treatment in 2M KOH at 80 °C, for the PBI-DIm and PBI-DIm-Si hybrid membranes.

The hydroxide conductivity of an AEM has a decisive influence on the fuel cell performance. In this work, we employed a 4-electrode method to conduct the hydroxide conductivity measurements for the OH^−^ form membranes under fully hydrated conditions. Generally, the hydroxide conductivity of AEM higher than 10^−2^ S cm^−1^ for better fuel cell performance is required [56]. As shown in Figure 6a,b, the measured hydroxide conductivity highly depended on temperature, obeying the Arrhenius law quite well. In the temperature range of 30 °C and 80 °C, the hydroxide conductivity of the PBI-DIm membrane increased from 1.52 × 10^−2^ S cm^−1^ to 5.02 × 10^−2^ S cm^−1^, whereas that of the PBI-DIm-Si hybrid membrane was measured varying from 6.36 × 10^−3^ S cm^−1^ to 2.22 × 10^−2^ S cm^−1^. Despite these two membranes having similar IECs, PBI-DIm exhibited relatively higher hydroxide conductivities than PBI-DIm-Si hybrid, which may be due to the morphology of hydrophilic/hydrophobic micro-phase separations that facilities the ion transport more effectively [20]. Moreover, the activation energy of hydroxide transport for the PBI-DIm and PBI-DIm-Si hybrid membranes can be obtained by means of the Arrhenius plots. The difference between their activation energies was found insignificant. This finding implied that the hydroxide transport mechanism was the same for these two membranes with different morphologies. It is known that the hydroxide can transport in AEMs via either the vehicular mechanism or the Grotthuss mechanism [57]. Previous studies suggested that the ion transport will obey vehicular mechanism if the activation energy is less than 14 kJ mol^−1^ [58,59]. Since the activation energies in these two membranes exceed 14 kJ mol^−1^, the Grotthuss mechanism is supposed to dominate the ion transport mechanism [60].

The alkaline stability of the AEMs based on dimethylimidazolium-functionalized PBI was evaluated by monitoring the change in hydroxide conductivity during the alkaline treatment which was conducted by immersing the membrane samples in 2 M KOH aqueous solution at 80 °C for 120 h. As shown in Figure 6c, there is almost no decay in hydroxide conductivity measured at 30 °C during the alkaline treatment for these two AEMs, indicating that they were alkaline stable and promising for practical applications in AEMFCs. The main reason may lie in the fact that the methyl-protecting groups at the C2-position of the imidazolium ring positively affect the alkaline stability of the membrane to prevent the ring-opening hydrolysis under strong alkaline conditions [61]. Recently, Dekel and his co-workers reported that lowing hydration number or water content would accelerate the degradation rate of cationic groups (e.g., benzyl trimethylammonium) and decrease the activation energy of hydroxide attack in the AEMs [62]. Accordingly, in our case, excess water in the vicinity of the cationic side groups (i.e., high λ) diluting the hydroxide ion concentration may weaken the nucleophilic attack of hydroxide ions to dimthylimidazolium, especially for the PBI-DIm membrane. Aside from these reasons, the crosslinked structure of the PBI-DIm-Si hybrid membrane may account for enhancing the membrane’s alkaline stability.

### 3.5. Mechanical Properties and Oxidative Stability

In general, ion conduction in AEMs under fully hydrated conditions functions better than under dry conditions. However, the excess water could impair the mechanical properties of AEMs, which is unfavorable for fabricating robust membrane electrode assemblies (MEAs). On the other hand, the gas diffusion electrode at the cathode undergoing the electrochemical reaction may generate lots of oxygen-related radicals during the fuel cell operation, which will attack the polymer backbone of AEM, leading to degradations [63,64]. Therefore, sufficient mechanical strength and adequate oxidative stability of AEMs are of importance for the long-term operation of fuel cells [65,66].

The mechanical properties of the PBI-DIm and PBI-DIm-Si hybrid membranes in the dry and fully hydrated states were summarized in Table 2. The results showed that the PBI-DIm-Si hybrid membrane retained 79.6% of its elastic modulus of the dry state (from 0.64 GPa to 0.51 GPa) when it reached a fully hydrated state. In contrast, the loss of elastic modulus was up to 71.4% (from 0.63 GPa to 0.18 GPa) for the PBI-DIm membrane. It is worth noting that both the tensile strength and elongation at break of the PBI-DIm-Si hybrid membrane in the fully hydrated state (28.2 MPa and 27.2%) were even higher than those of the PBI-DIm membrane in the dry state (26.0 MPa and 15.2%). This result revealed that the organic–inorganic hybrid network effectively enhanced the mechanical property of the AEM based on side chain type quaternized PBI. Additionally, the tensile strength values of these two membranes in the fully hydrated state were comparable with the reported value of Nafion 112 (19.1 MPa) [67] and other quaternized PBI membranes [24,68,69], suggesting that they were mechanically strong enough for fuel cell applications.

Furthermore, it is worth noting that all the tensile properties in terms of elastic modulus, tensile strength did not differ significantly before and after the alkaline treatment in 2 M KOH solution at 80 °C for 120 h, whether for the PBI-DIm or PBI-DIm-Si hybrid membranes in the dry state (Table 2). This result confirmed that the PBI polymer backbone was alkaline stable, as mentioned earlier.

**Figure 7:**
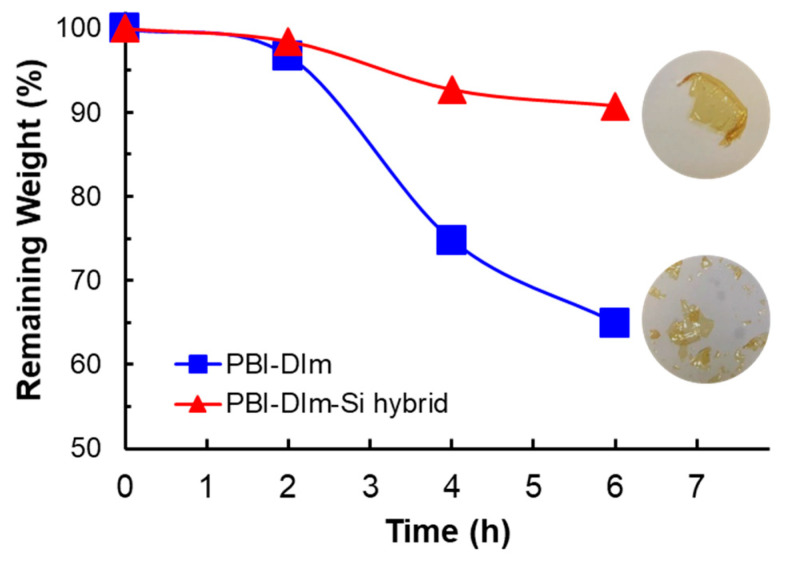
Oxidative stability of the PBI-DIm and PBI-DIm-Si hybrid membranes.

To evaluate the oxidative stability of the PBI-DIm and PBI-DIm-Si hybrid membranes, they were immersed in an aqueous solution containing Fenton agents (3 wt.% H_2_O_2_ and 4 ppm Fe^2+^) full of radicals at 80 °C for a period of time. Figure 7 shows the remaining weight of the membrane sample as a function of time during the Fenton test. It was found that the remaining weight of the BI-DIm-Si hybrid membrane was 90.7 wt.% after 6 h, which was much higher than that of the PBI-DIm membrane (64.9 wt.%). This result indicated that the organic–inorganic hybrid allowed the quaternized PBI to improve the resistance to the radical attack and reduce chemical degradation efficiently.

### 3.6. Fuel Cell Performance

**Figure 8:**
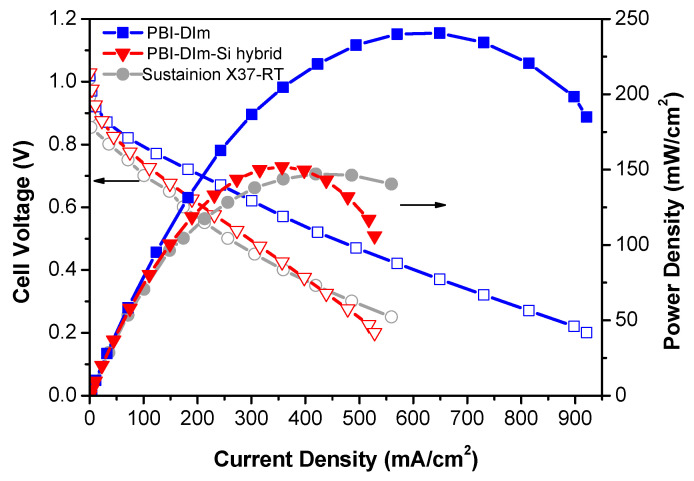
Fuel cell performance for the MEA based on the PBI-DIm, PBI-DIm-Si hybrid, and commercially available AEM Sistainion X37-RT.

In order to confirm the applicability of the PBI-DIm and PBI-DIm-Si hybrid membranes in AEMFCs, the fuel cell performance of their MEAs was examined and further compared with that of the commercially available AEM Sistainion X37-RT. Figure 8 shows the polarization curves of the MEAs conducted under humidified hydrogen (50 mL min^−1^) and oxygen (100 mL min^−1^) atmosphere at 60 °C without back-pressure. The open-circuit voltages (OCVs) of fuel cells for these AEMs were detected in the range of 1.01 V and 1.03 V, indicating that the gas crossover problem was insignificant. The maximum power density of the PBI-DIm membrane reached up to 241 mW cm^−2^, which was higher than that of Sistainion X37-RT (147 mW cm^−2^) as well as the reported values of other commercially available AEMs Fumatech FAA-3 (223 mW cm^−2^) and Tokuyama AHA (40.2 mW cm^−2^) [70,71]. The fuel cell performance of the MEA based on the PBI-DIm-Si hybrid membrane was found to be not as good as that of the PBI-DIm membrane, which would result from its relatively lower hydroxide conductivity (1.32 × 10^−2^ S cm^−1^ at 60 °C). Despite that, it achieved the maximum power density of 152 mW cm^−2^, which was comparable to the performance of the MEA based on Sistainion X37-RT. These results suggested that both the pristine and organic–inorganic hybrid membranes of dimethylimidazolium-functionalized PBI are promising for fuel cell applications.

## 4. Conclusions

In this work, the silane-induced crosslinking of the side chain type BPI was studied. The pristine and organic–inorganic hybrid membranes of quaternized PBIs with side chains containing dimethylimidazolium groups were successfully prepared and characterized. The experimental results confirmed that the silane-induced crosslinking networks can improve thermal stability, dimensional stability, mechanical strength, and oxidative stability of the AEMs based on dimethylimidazolium-functionalized PBI. The PBI-DIm pristine membrane successfully developed a membrane morphology of distinguishable hydrophilic/hydrophobic phase separation. However, the phase separation was no longer clear when many silica-based domains appeared in the PBI-DIm-Si hybrid membrane, evidenced by AFM phase image mapping, SAXS diffraction, and XPS spectra deconvolution. Both membranes exhibited sufficient hydroxide conductivity (>10^−2^ S cm^−1^) and good alkaline stability in 2 M KOH at 80 °C. Meanwhile, the power densities of the MEAs based on these two membranes were higher than that of commercially available AEM Sistainion X37-RT (147 mW cm^−2^), demonstrating the promising potential of these two AEMs used in AEMFCs. Their long-term durability of fuel cell performance is worthy of evaluation in future work.

## Figures and Tables

**Table 1:** Properties of the PBI-DIm and PBI-DIm-Si hybrid membranes.

AEM	Theoretical IEC ^1^ (mmol/g)	Experimental IEC ^4^ (mmol/g)	WU ^5^ (%)	SR ^5^ (%)	λ	Insoluble Solid Content (wt%)
PBI-DIm	2.02 ^2^	1.85	62.7	26.7	18.8	62.4
PBI-DIm-Si hybrid	2.05 ^3^	1.87	30.9	10.0	9.2	78.8

^1^ determined for the Cl^−^ form membrane. ^2^ calculated from the functionalization degree of PBI-DIm. ^3^ calculated from the molar ratio of CPTMOS: [SPDMIm] [Cl]: PBI-DIm. ^4^ measured by Mohr titration for the Cl^−^ form membrane. ^5^ determined for the OH^−^ form membrane.

**Table 2:** Mechanical properties of the PBI-DIm and PBI-DIm-Si hybrid membranes.

State	AEM	Elastic Modulus (GPa)	Tensile Strength (MPa)	Elongation at Break (%)
Dry	PBI-DIm	0.63 ± 0.14	26.0 ± 3.5	15.2 ± 3.6
	PBI-Dim-Si hybrid	0.64 ± 0.11	32.9 ± 3.7	16.9 ± 4.2
Fully hydrated	PBI-DIm	0.18 ± 0.04	17.0 ± 0.7	31.9 ± 4.0
	PBI-Dim-Si hybrid	0.51 ± 0.04	28.2 ± 2.5	27.2 ± 4.83
Dry, alkaline degraded ^1^	PBI-DIm	0.64 ± 0.12	25.8 ± 3.8	15.9 ± 3.5
	PBI-Dim-Si hybrid	0.66 ± 0.09	33.1 ± 4.1	18.3 ± 4.5

^1^ The membrane samples were immersed in 2 M KOH at 80 °C for 120 h before drying and subsequently tensile testing.
